# High sample throughput genotyping for estimating C-lineage introgression in the dark honeybee: an accurate and cost-effective SNP-based tool

**DOI:** 10.1038/s41598-018-26932-1

**Published:** 2018-06-04

**Authors:** Dora Henriques, Keith A. Browne, Mark W. Barnett, Melanie Parejo, Per Kryger, Tom C. Freeman, Irene Muñoz, Lionel Garnery, Fiona Highet, J. Spencer Jonhston, Grace P. McCormack, M. Alice Pinto

**Affiliations:** 1 Mountain Research Centre (CIMO), Polytechnic Institute of Bragança, 5300-253 Bragança, Portugal; 2 Centre of Molecular and Environmental Biology (CBMA), University of Minho, Campus de Gualtar, 4710-057 Braga, Portugal; 3 Department of Zoology, Ryan Institute, School of Natural Sciences, National University of Ireland Galway, Galway, Ireland; 4 The Roslin Institute and Royal (Dick) School of Veterinary Studies, University of Edinburgh, Easter Bush, Edinburgh, Midlothian, EH25 9RG Scotland UK; 5 Agroscope, Swiss Bee Research Centre, 3003 Bern, Switzerland; 6 Aarhus University, Department of Agroecology, Slagelse, 4200 Denmark; 7 Área de Biología Animal, Dpto. de Zoología y Antropología Física, Universidad de Murcia, Campus de Espinardo, 30100 Murcia, Spain; 8 Laboratoire Evolution, Génomes et Spéciation, CNRS, Gif-sur-Yvette, France; 9 Saint Quentin en Yvelines, Université de Versailles, Versailles, France; 10 Science and Advice for Scottish Agriculture (SASA), Roddinglaw Road, Edinburgh, EH12 9FJ Scotland UK; 11 Department of Entomology, Texas A&M University, College Station, USA

## Abstract

The natural distribution of the honeybee (*Apis mellifera* L.) has been changed by humans in recent decades to such an extent that the formerly widest-spread European subspecies, *Apis mellifera mellifera*, is threatened by extinction through introgression from highly divergent commercial strains in large tracts of its range. Conservation efforts for *A*. *m*. *mellifera* are underway in multiple European countries requiring reliable and cost-efficient molecular tools to identify purebred colonies. Here, we developed four ancestry-informative SNP assays for high sample throughput genotyping using the iPLEX Mass Array system. Our customized assays were tested on DNA from individual and pooled, haploid and diploid honeybee samples extracted from different tissues using a diverse range of protocols. The assays had a high genotyping success rate and yielded accurate genotypes. Performance assessed against whole-genome data showed that individual assays behaved well, although the most accurate introgression estimates were obtained for the four assays combined (117 SNPs). The best compromise between accuracy and genotyping costs was achieved when combining two assays (62 SNPs). We provide a ready-to-use cost-effective tool for accurate molecular identification and estimation of introgression levels to more effectively monitor and manage *A*. *m*. *mellifera* conservatories.

## Introduction

Pollination by the honeybee (*Apis mellifera* L.) is a blended ecosystem service of managed and unmanaged (feral or wild) colonies that is under threat from human-mediated environmental changes including climate change, habitat loss, habitat fragmentation, pesticides, and introduced parasites and pathogens^1,2^. There is growing evidence that management of locally adapted genetic diversity in honeybee subspecies and ecotypes is key to the long-term sustainability of this service^3–5^. Accordingly, actions towards preserving the large stores of genetic diversity held by the 31 honeybee subspecies^6–9^ are expected to counteract the trend of global colony losses.

Of the 31 subspecies that have been identified in the natural distributional range of *A*. *mellifera* in Africa, Middle East, Western Asia, and Europe^6,9,10^ there are 10 European subspecies grouped into two evolutionary lineages^10^: the Western and Northern European (lineage M) and the South-eastern European (lineage C). Lineage M includes only two subspecies: the Dark honeybee *Apis mellifera mellifera* and the Iberian honeybee *Apis mellifera iberiensis*. Yet, these two subspecies cover the largest territory in Europe with *A*. *m*. *iberiensis* occupying the Iberian Peninsula and *A*. *m*. *mellifera* ranging from France in the south to Scandinavia in the north, and from Ireland and the UK in the west to the Ural Mountains in the east^10^. Lineage C occurs in a smaller geographical area composed of the Apennine and Balkan peninsulas and includes the most widely kept honeybee subspecies: the Italian *Apis mellifera ligustica* and the Carniolan *Apis mellifera carnica*. In spite of its wide distribution, *A*. *m*. *mellifera* is the subspecies most under threat as it is considered extinct in many parts of Europe not only because of the human-mediated environmental changes but more insidiously through replacement by and introgression from non-indigenous subspecies, particularly *A*. *m*. *ligustica* and *A*. *m*. *carnica*^11–13^.

It has been argued that, unlike with other domesticated stock organisms, management and selective breeding in honeybees increase genetic diversity through introgression^14^. However, this form of admixture reduces the frequency of locally adapted gene complexes, leading to an increased likelihood of reduced survival rates of colonies^15^. How to protect locally adapted gene complexes that are more suited to local environments is a growing problem, as the increased breeding and movement of C-lineage honeybees promotes sympatry and gene flow between *A*. *m*. *mellifera* and imported commercial breeds. Efforts to assist conservation of *A*. *m*. *mellifera* are gathering momentum in multiple European countries (www.sicamm.org) and with the knowledge that reduced adapted genetic diversity threatens both managed and unmanaged populations, the interests of commercial beekeeping and honeybee conservationists should be aligning, particularly in *A*. *m*. *mellifera* indigenous areas.

An important first step in protecting *A*. *m*. *mellifera* populations in official or unofficial conservatories is to give the stakeholders an accurate and cost-efficient tool to test for C-lineage introgression. Microsatellites have been extensively used to examine C-lineage introgression in *A*. *m*. *mellifera*^11,12,16^. Yet, the numerous advantages of SNPs over microsatellites promise to make them the tool of choice for population monitoring and conservation purposes. In addition to being more abundant and widespread in the genome^17^, SNPs display lower genotyping error, have higher quality data, are more amenable to automated analysis and data interpretation, and can be easily transferred between laboratories^18^. Moreover, SNPs proved to be more powerful than microsatellites at estimating C-lineage introgression in *A*. *m*. *mellifera*^19^. These properties make SNPs a powerful tool for testing the breeding stock in *A*. *m*. *mellifera* conservatories and SNP data can be readily incorporated in shared genetic databases, facilitating implementation of a conservation strategy at the European scale.

Whilst SNP analysis on whole genome (WG) sequence data may be required in studies concerned with fine-scale relatedness, such deep sequencing is disproportionate when determining introgression levels for the discrimination of *A*. *m*. *mellifera* breeding stocks. Also, while costs have dropped dramatically, WG sequencing is still unaffordable for most stakeholders committed to the long-term sustainability and conservation of honeybees. Costs are accrued as WG analysis requires considerable computing storage and processing power and trained bioinformatics personnel. However, encouragingly, Muñoz, *et al*.^20^ showed that reduced panels of highly informative SNPs can accurately identify honeybee stocks^20–23^. Genotyping using reduced SNP panels considerably decreases laboratory processing costs. Furthermore, analysis of the generated genotypes requires low computational power and conventional bioinformatics skills.

Muñoz, *et al*.^20^ developed reduced SNP panels for genetic identification and introgression analysis in *A*. *m*. *mellifera*. The authors used a combination of metrics to rank by information content over 1183 SNPs that had been genotyped in *A*. *m*. *mellifera*, *A*. *m*. *ligustica* and *A*. *m*. *carnica* using the 1536-plex GoldenGate® Assay of Illumina^13^. The top-ranked SNPs were combined into five nested panels whose sizes (48, 96, 144, 192, 384 SNPs each^20^) fitted the plexes of the now discontinued GoldenGate® Assays formerly genotyped with the VeraCode® technology. Here, we built from the 144-SNP panel to propose four customized assays tailored for high sample throughput genotyping using the iPLEX MassARRAY system. By providing a ready-to-use molecular tool for accurately, rapidly, and cost-effectively genotyping large sample sizes of *A*. *m*. *mellifera*, we hope to bring affordable C-lineage introgression detection to stakeholders in the fight to safeguard remaining reservoirs of unique combinations of genes and adaptations in *A*. *m*. *mellifera* and to expand its reduced current distribution.

## Results

### Assay design, quality control and genotyping accuracy

**Figure 1:**
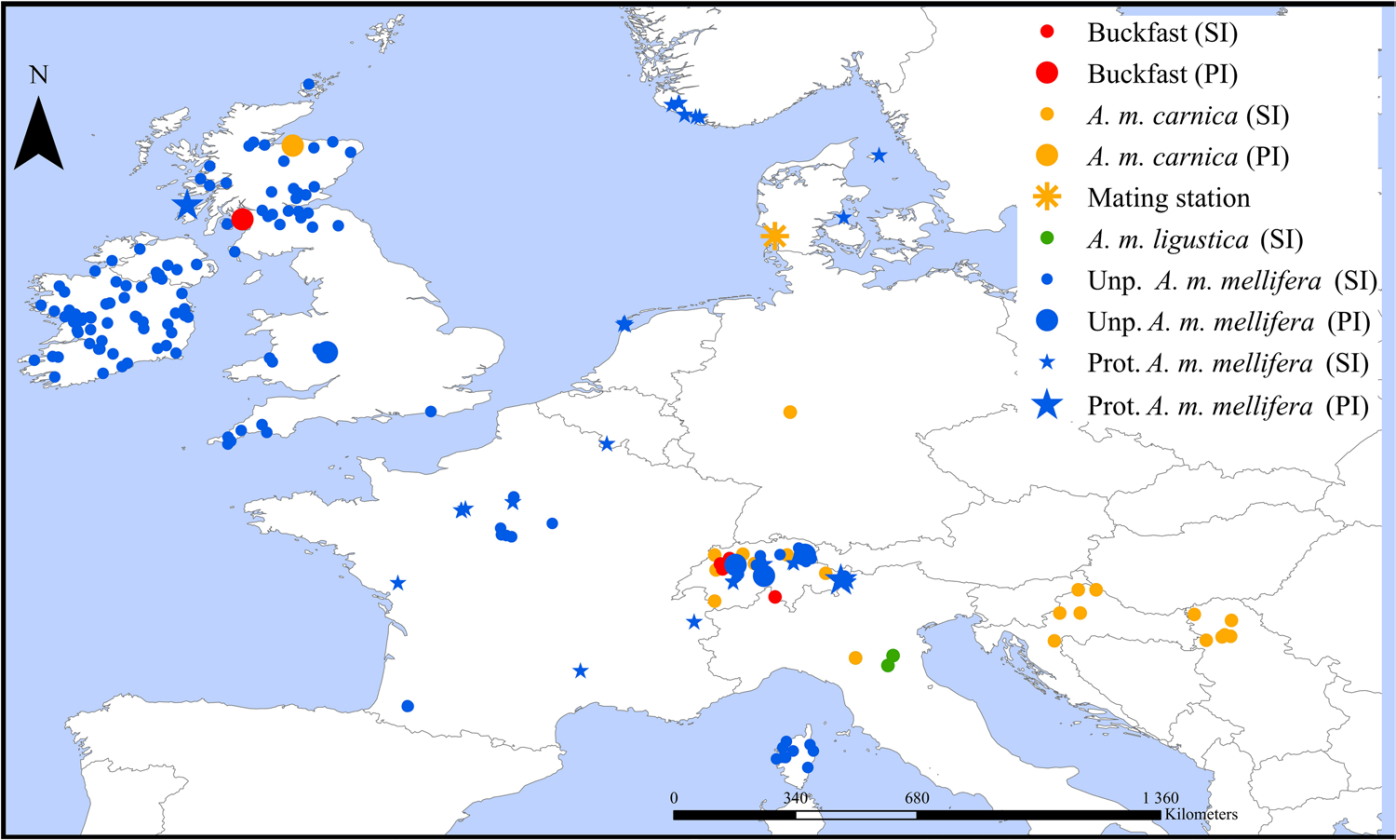
Location of the colonies sampled across the *A*. *m*. *mellifera* and C-lineage ranges. Samples of *A*. *m*. *mellifera* were collected in protected (Prot) and unprotected apiaries (Unp). The commercial breed Buckfast is also represented. Colonies were genotyped for the four SNP assays in the MassARRAY® MALDI-TOF platform from single individuals (SI) or pools of individuals (PI).

Of the 144 highly-informative SNPs selected by Muñoz, *et al*.^20^, the Assay Design software was able to multiplex 127 into four assays (identified by letter M), each containing a variable number of SNPs ranging from 38 in M1 to 24 in M4 (Supplementary Table S1). A total of 573 samples (Fig. 1) were genotyped for the four customized assays using the iPLEX MassARRAY system. Of the 573 samples, only seven displayed a SNP call failure rate >30% and these were excluded from further analysis (Supplementary Table S2). Of the 566 remaining samples, 551 displayed a low percentage (<10%) of missing data indicating a high genotyping success rate (96%).

**Figure 2:**
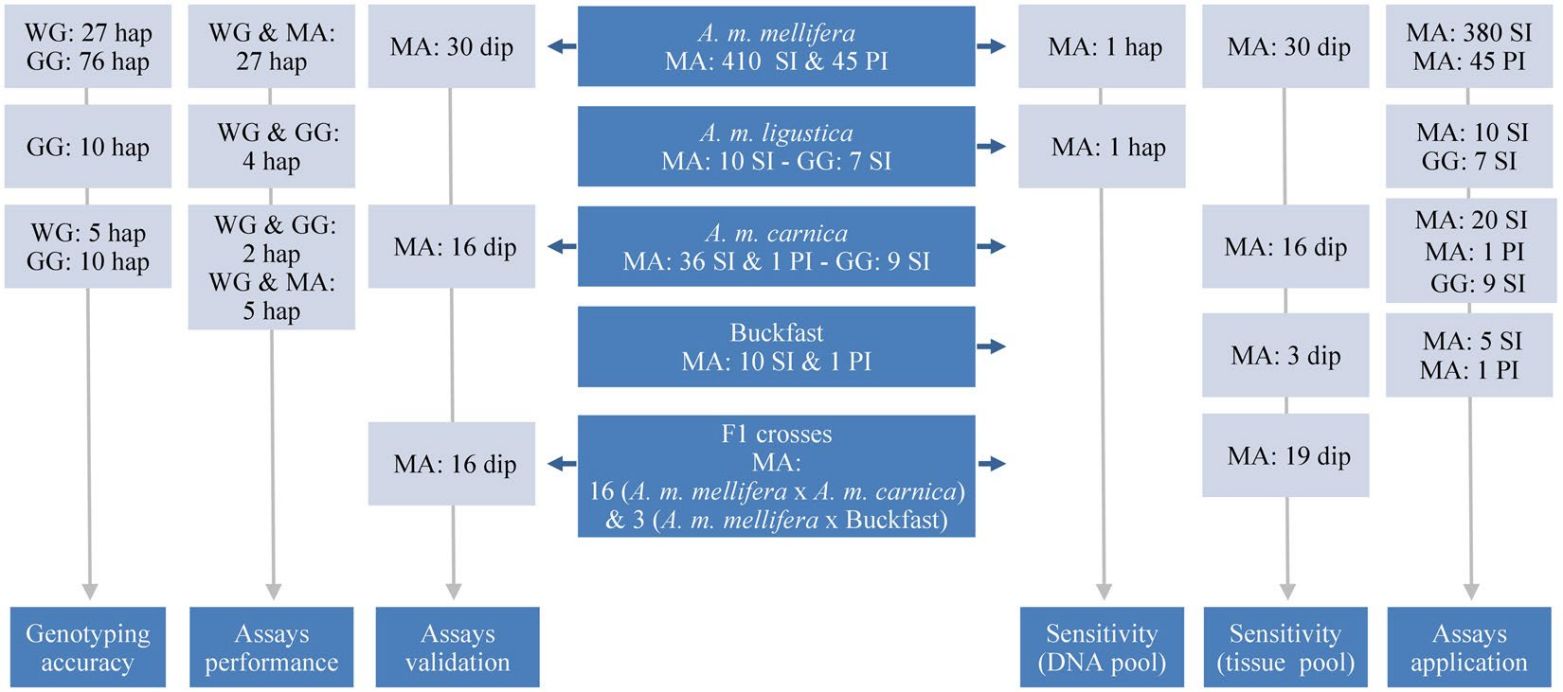
Datasets of quality-proved samples used in the SNP assays’ testing and application. Samples were represented by a single individual (SI) or a pool of individuals (PI). The individuals were haploid drones (hap) or diploid workers (dip). Genotypes were generated from the four assays in the MassARRAY® MALDI-TOF platform (MA), from the GoldenGate® Assay in the Illumina’s BeadArray platform (GG), and from whole genome (WG) sequences in the Illumina’s HiSeq. 2500 platform.Vertical arrows connect the different individuals used in each test.

The quality control and assessment of the genotyping accuracy of the 127 SNPs (Supplementary Table S3) led to identification of 10 problematic SNPs, of which seven were typed in <80% of the individuals, three were called heterozygous for >10% of the haploid individuals (Supplementary Tables S1 and S3), and three exhibited inconsistent calls among the three genotyping technologies in >5% of the individuals (Fig. 2). The latter SNPs were also identified as having high rates of missing data or heterozygosity (Supplementary Table S3). Once the 10 SNPs were removed from the datasets, the rates of missing data of the remaining 117 SNPs were low with 113 having <10% and four varying between 10.4% and 15.5% (Supplementary Table S1). The genotypes generated for the 117 high-quality SNPs in the MassARRAY platform were highly concordant with those of the Illumina’s platforms (99.9% for the BeadArray and 99.6% for the HiSeq. 2500). Following the quality control step, 339 of the 573 genotyped samples had no missing data and the highest rate of missing data was 29% but only in two samples (Supplementary Table S2).

**Figure 3:**
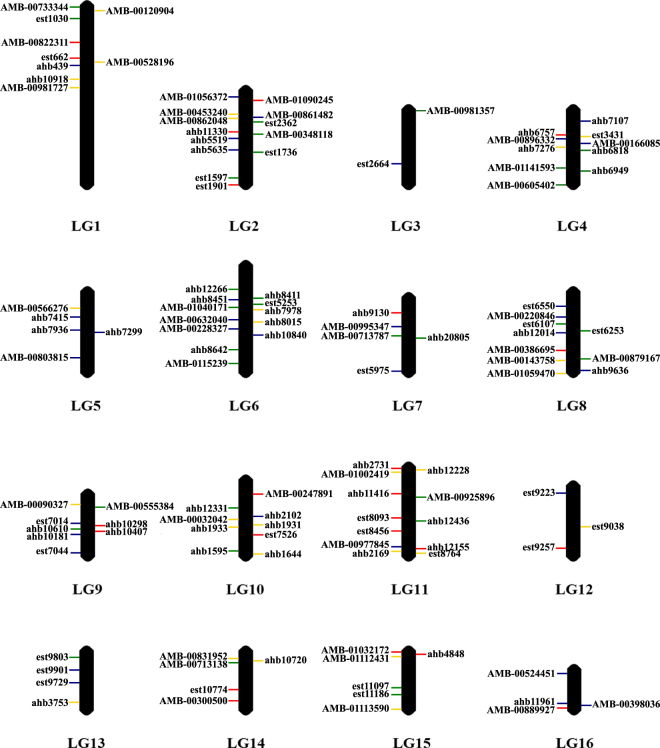
Genomic positions of the 117 quality-proved SNPs. The 117 SNPs were multiplexed in four assays, named M1 (blue), M2 (green), M3 (yellow), and M4 (red).

The final multiplexes contained M1 = 34, M2 = 32, M3 = 28, and M4 = 23 SNPs distributed across the 16 honeybee linkage groups, LGs (Fig. 3 and Supplementary Tables S1 and S4). LG 2 harboured the highest number of SNPs (13) while LG 3 had the lowest (2). The number of LGs covered by the assays varied between 12 (M4) and 14 (M1). Most SNPs (90 of 117) are located in non-coding regions, including intergenic (50 SNPs), intronic (30 SNPs), and UTRs (10 SNPs). Of the 27 coding SNPs, only two (1384-est6107 and 661-AMB-00398036) are non-synonymous (Supplementary Table S1).

### Assessing performance of the SNP assays

The performance of the four assays was assessed by comparing their *Q*-values (inferred from single or combined assays) with those inferred from the genome-wide SNPs, which provides the best estimate of the admixture proportions (Supplementary Table S5). The four assays exhibited a good individual performance with a mean accuracy >94% and *Q*-values highly correlated (0.980 ≤ *r* ≤ 0.983) with those inferred from the WG dataset (Table 1). The largest plex assay M1 (34 SNPs) and the smallest M4 (23 SNPs) showed the best and the worst behaviour, respectively, as indicated by most statistics (Table 1). The best performance was achieved when the four assays (117 SNPs) were used together (*r* = 0.996; mean accuracy = 97.84%; absolute precision error = 0.033), although the combination of M1 + M3 (62 SNPs) and M1 + M2 + M3 (94 SNPs) with the highest individual correlations produced equally interesting statistics with mean accuracies >96.9%, absolute precision error <0.04, and with over 28 individuals (out of 32) with absolute accuracy error <0.05. Performance was also assessed by counting purebred *A*. *m*. *mellifera* individuals misclassified as admixed (*Q*-values > 0.05) and *vice versa* (Table 1). Except for M4, single assays and their combinations repeatedly misclassified two or three (always identified amongst individuals M23, M24, M25, and M26; Supplementary Table S5) purebred as admixed from 11 *A*. *m*. *mellifera* individuals identified by genome-wide SNPs. The degree of *A*. *m*. *mellifera* misclassification was lower for the class “admixed identified as purebred” with M3, and its combination with one (M1), two (M1 + M2) or three assays (M1 + M2 + M4) correctly identifying all 16 admixed individuals (0.05 < *Q*-value < 0.95).

**Table 1:** Statistics for the performance of the four SNP assays used singly or combined.

SNP Assay	# of SNPs	(i)	(ii)	(iii)	(iv)	(v)	(vi)	(vii)	(viii)	(ix)
M1	34	0.983	0.929	0.046	0.211	26	95.42	0.061	2	1
M2	32	0.981	0.919	0.051	0.239	24	94.86	0.068	3	2
M3	28	0.982	0.926	0.047	0.314	23	95.27	0.066	3	0
M4	23	0.980	0.911	0.050	0.283	23	95.00	0.067	0	2
M1 + M3	62	0.993	0.956	0.029	0.172	31	97.09	0.042	2	0
M1 + M2 + M3	94	0.994	0.957	0.031	0.137	28	96.94	0.040	3	0
M1 + M2 + M3 + M4	117	0.996	0.964	0.022	0.114	32	97.84	0.033	2	0

Calculations were made via comparisons between *Q*-values inferred from the SNP assays and the genome-wide 2.399 million SNPs. (i) Pearson’s correlation coefficient (*r*); (ii) similarity score obtained by CLUMPAK; (iii) mean and (iv) maximum absolute accuracy errors; (v) number of individuals (out of 38) with absolute accuracy error <0.05; (vi) mean accuracy estimated via percentage of absolute error; (vii) absolute precision error; (viii) number of purebred *A*. *m*. *mellifera* individuals misclassified as admixed; (ix) number of admixed individuals misclassified as purebred.

### Validating the SNP assays

**Figure 4:**
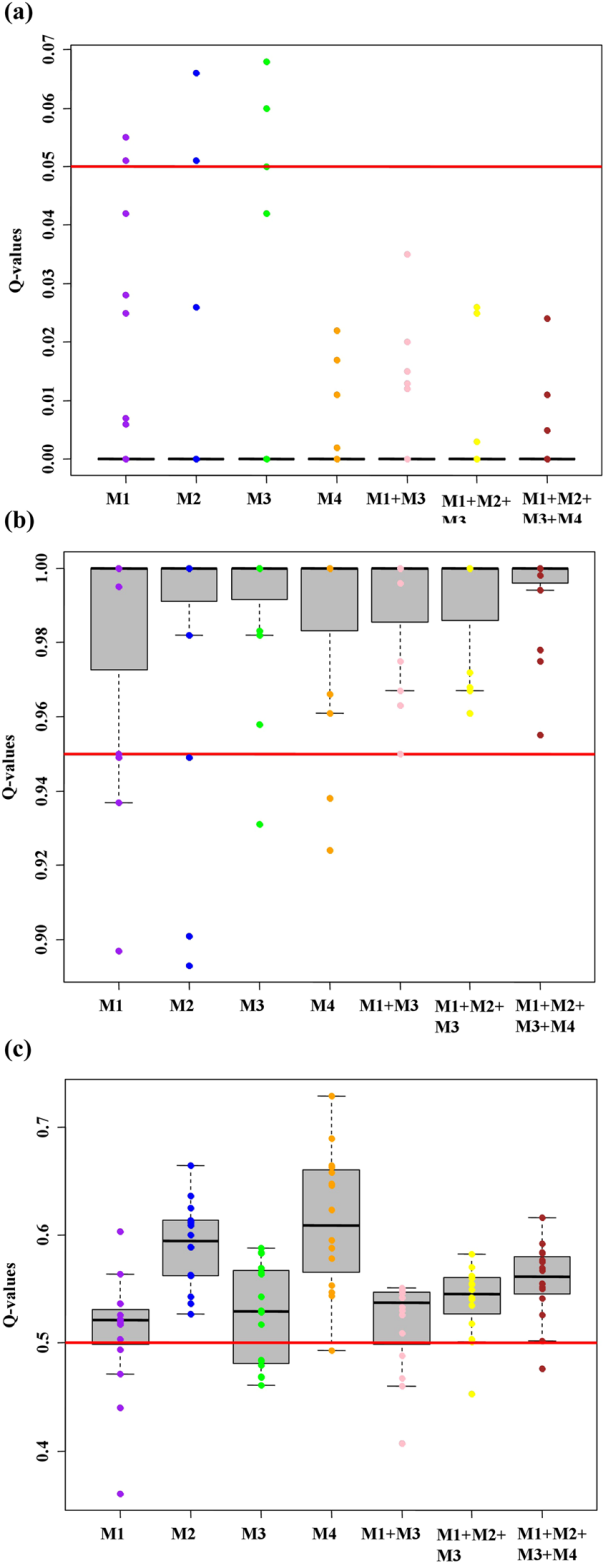
Validating the four SNP assays. Boxplots showing the variation of the *Q*-values inferred from the called genotypes for the four SNP assays. The boxes denote the first and third quartiles. The horizontal red lines mark the expected *Q*-values for purebred *A*. *m*. *mellifera and A*. *m*. *carnica* set at <0.05 and >0.95, respectively, and for the F1 hybrid samples set at 0.5. Boxplots for the (**a**) 30 *A*. *m*. *mellifera* samples, (**b**) 16 *A*. *m*. *carnica* samples, and (**c**) 16 F1 hybrid samples.

The assays were validated using an independent set of 62 individuals, including 30 *A*. *m*. *mellifera*, 16 *A*. *m*. *carnica*, and 16 F1 hybrids. On average, *Q*-values inferred from the genotypes called using the four individual (M1, M2, M3, M4) and three combined assays (M1 + M3, M1 + M2 + M3, M1 + M2 + M3 + M4) fit the thresholds defined for the two subspecies and hybrids (*P*-value ≥ 0.18, Mann-Whitney test; Supplementary Table S6). Despite good overall performance of the individual assays, a few purebred *A*. *m*. *mellifera* and *A*. *m*. *carnica* were misclassified as admixed (estimated *Q*-values deviated from thresholds of <0.05 for *A*. *m*. *mellifera* and >0.95 for *A*. *m*. *carnica*) when the *Q*-values were inferred from called genotypes. However, when mixed combinations of the four assays were employed, the estimated *Q*-values matched the expectations with all *A*. *m*. *mellifera* and *A*. *m*. *carnica* correctly classified as purebred (Fig. 4a,b) and the F1 hybrids varying between 0.52 ± 0.04 (mean ± SD), for M1 + M3, and 0.56 ± 0.03, for the four assays combined, with the slight bias toward the *A*. *m*. *carnica* in the F1 (Fig. 4c) reflecting the known low level of C-derived introgression in the Læsø source population^13^.

### Assessing sensitivity of the MassARRAY system in pooled DNA

**Figure 5:**
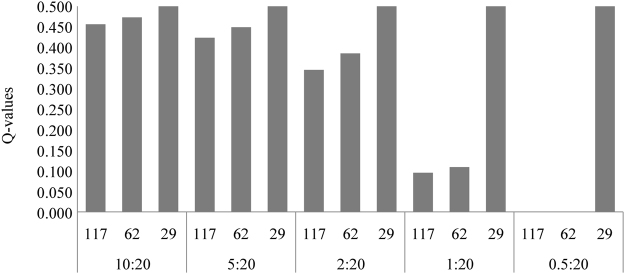
Average *Q*-values for different DNA pools. *Q*-values were inferred for DNA pools (representing dilution ratios of 10:20, 5:20, 2:20, 1:20, 0.5:20) by the four SNP assays (117 SNPs), the two best assays M1 + M3 (62 SNPs) and the 29 SNPs that were identified in all dilution ratios. The *Q*-values of the four SNPs assays and the M1 + M3 decreased as the dilution ratios increased. The *Q*-values of the 29 SNPs were always 0.50, as these were all heterozygous.

The sensitivity of the MassARRAY system in detecting *A*. *m*. *ligustica* was assessed in pools combining the DNA of two haploid individuals (one *A*. *m*. *mellifera* and one *A*. *m*. *ligustica*) at five dilution ratios. Of the 117 SNPs, only 103 were informative in this experiment (five were monomorphic, and nine were bi-allelic, but only one allele was called across dilutions). As expected, the sensitivity decreased as the dilution ratios increased, with only 29 unlinked SNPs being able to detect the *A*. *m*. *ligustica* alleles in every dilution and replicate (Supplementary Fig. S1, Supplementary Information). Yet, it was still possible to detect introgression with either the four assays (117 SNPs) or the two assays M1 + M3 (62 SNPs), even when the *A*. *m*. *ligustica* DNA was as diluted as 1:20 (Fig. 5 and Supplementary Table S7).

### Assessing sensitivity of the MassARRAY system in pooled tissue

The sensitivity of the MassARRAY was further assessed in 22 tissue pools. Of the 2,574 called genotypes (117 SNP loci × 22 pools), 1,977 (77%) were accurate, as determined by comparing the calls for single workers with those of the pools. The most common sources of mismatch were “the most frequent allele” and “higher DNA concentration” (Table 2). The average rate of accurately called SNPs per pool was high (77%, 90 SNPs) and varied between 83% (97 SNPs), for the pools of two workers, and 50% (58 SNPs), for the pools of eight workers (Table 3).

**Table 2:** Information on SNP calling obtained from the 22 tissue pools.

SNP calling	# of genotypes
Sources of allele miscalling	
Different alleles	5
Higher DNA concentration	86
Higher DNA concentration & the most frequent allele	81
The most frequent allele	279
The least frequent allele	42
Missing data	104
Accurate calls	1,977
Total	2,574

**Table 3:** Mean number of SNP loci accurately called and miscalled for the different combination of tissue pools.

Tissue pools (# of replicates)	Accurate SNPs	Miscalled SNPs				
		i	ii	iii	iv	v
1 Mel + 1 Hyb (3)	97.0	1.0	4.7	4.7	5.3	0.3
2 Mel + 1 Hyb (2)	81.0	0.0	3.5	5.0	21.0	0.5
3 Mel + 1 Hyb (2)	83.5	0.0	5.5	5.0	17.5	0.0
7 Mel + 1 Hyb (2)	58.0	0.0	4.5	5.0	47.0	0.5
1 Mel + 1 Car (3)	85.0	0.3	4.3	4.0	5.3	11.0
1 Car + 1 Hyb (3)	101.7	0.3	3.7	2.0	4.0	1.3
2 Car + 1 Hyb (2)	93.5	0.0	3.0	2.5	13.0	0.0
3 Car + 1 Hyb (2)	93.0	0.0	3.0	5.0	12.0	0.0
1 Buc + 1 Hyb (3)	102.7	0.0	3.0	1.3	4.7	0.7

The sources of miscalling were (i) different alleles, (ii) higher DNA concentration, (iii) higher DNA concentration and the most frequent allele, (iv) the most frequent allele, and (vi) the least frequent allele. Mel - *A*. *m*. *mellifera*; Hyb – F1 hybrid; Car – *A*. *m*. *carnica*; Buc – Buckfast.

The *Q*-values estimated for the 22 pools from the called genotypes were similar to those estimated from the expected genotypes using either the four assays, M1 + M3, or the 29 SNPs (*P*-value ≥ 0.35, Mann-Whitney test). Furthermore, the MassARRAY platform was able to detect low frequency alleles, either of M-lineage (pools containing *A*. *m*. *mellifera*) or C-lineage ancestry (pools containing *A*. *m*. *carnica* or Buckfast), even when the tissue dilution was as low as 1:7 (Supplementary Table S8).

### Applying the SNP assays

**Figure 6:**
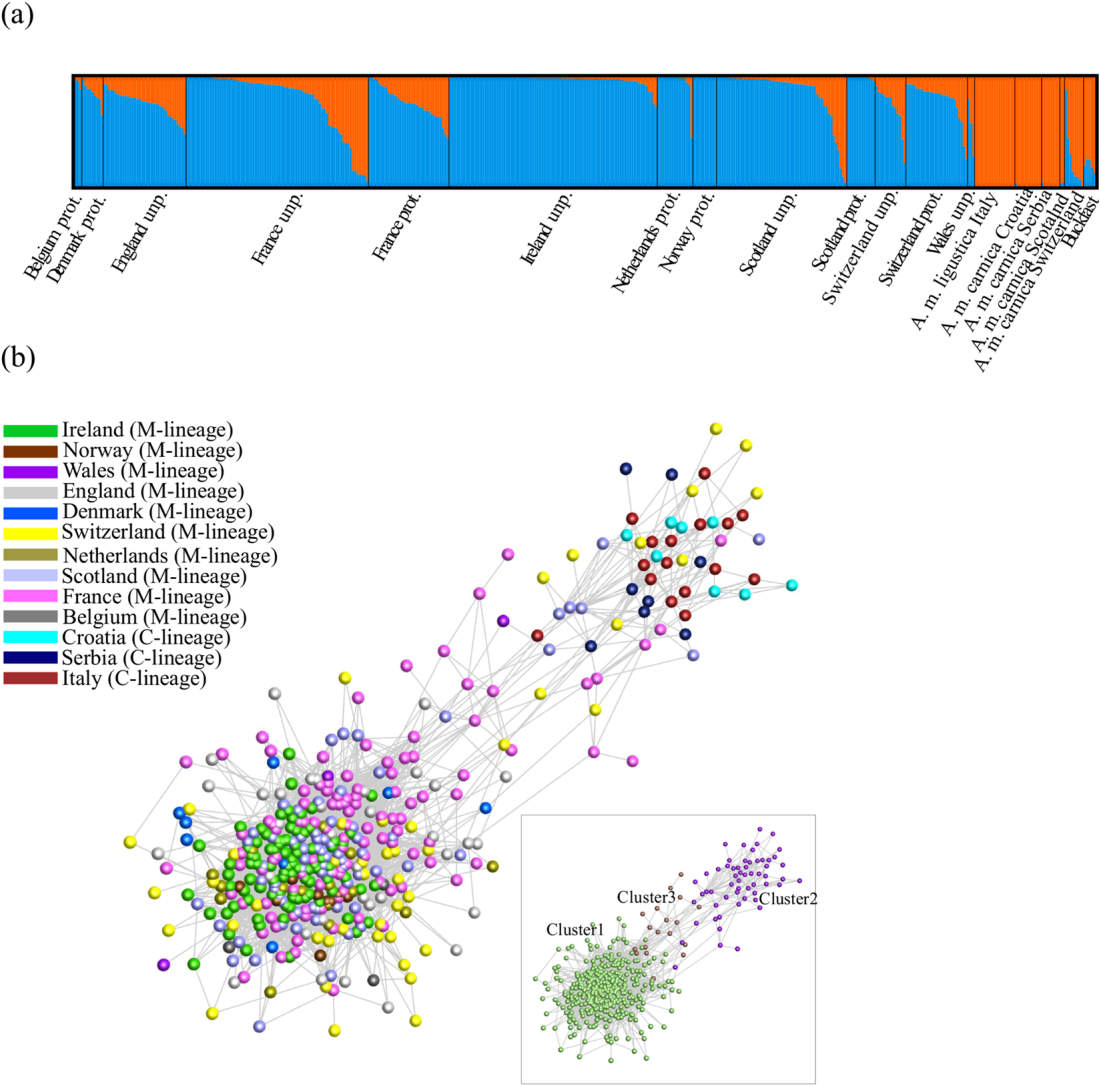
Structure reconstructed by ADMIXTURE and Graphia Professional software packages for honeybees of diverse ancestry collected across Europe. Most depicted samples (415) were genotyped in the MassARRAY platform using the four assays (117 SNPs). Nine samples of *A*. *m*. *carnica* and seven *A*. *m*. *ligustica*, previously genotyped for the 117 SNP loci using the GoldenGate Assay in the BeadArray platform, were added to the structure analysis for a better representation of C-lineage diversity. Each sample corresponds to a single colony. Samples collected in the *A*. *m*. *mellifera* range are from protected (prot) and unprotected (unp) apiaries. (**a**) ADMIXTURE plot showing the genome partitioning into two clusters (K = 2) for each individual, represented by a vertical bar. Blue represents the *A*. *m*. *mellifera* cluster and orange the C-lineage cluster. The black lines separate individuals from different countries and studied groups. (**b**) Correlation network where nodes (honeybee samples) are connected with edges when *r* > 0.27. A total of 418 samples out of 431 formed connections in the graph. Samples coloured according to country of origin with expected lineage indicated within parentheses. Inset shows correlation network clustered using the Markov Cluster (MCL) algorithm at an inflation value of 1.2.

The four assays were applied to 478 colonies of various ancestries, represented by single (431 colonies) or pooled individuals (47 colonies), collected in 13 European countries (Fig. 1). The *Q*-values estimated for each *A*. *m*. *mellifera* colony (Fig. 6a), indicated that introgression varies throughout Europe, ranging on average from 0.0 in Norway to 0.447 ± 0.265 in Wales (Supplementary Table S9). The least introgressed *A*. *m*. *mellifera* colonies were from conservatories of Norway (0 ± 0.000), Scotland (0.006 ± 0.011) and Netherlands (0.046 ± 0.141) with over 80% of the individuals showing a *Q*-value < 0.05, although most individuals (91%) of the unprotected populations of Ireland were also very pure (0.021 ± 0.022). Populations of Denmark, France and Switzerland exhibited greater *Q*-values (0.148 ≤ *Q*-value ≤ 0.280) in both protected and unprotected populations with ≤11% of pure individuals. Admixture proportions estimated for *A*. *m*. *ligustica* and *A*. *m*. *carnica* sampled from native and introduced ranges showed that they are very pure (0.972 ≤ *Q*-value ≤ 1.000), excepting for some Swiss colonies (0.750 ± 0.296). The commercial breed Buckfast was mostly of C-derived ancestry (0.806 ± 0.055).

The genotype data were further examined by network analysis. The correlation network graph shown in Fig. 6b consisted of 5,522 edges and 418 nodes (samples). Samples with similar allele profiles clustered together. In total, three clusters were identified with cluster 1 containing 342 nodes (highest similarity to M-lineage), cluster 2 containing 58 nodes (highest similarity to C-lineage) and cluster 3 containing 18 nodes (highest rates of introgression). All samples from Norway, Ireland, Netherlands and Belgium were in cluster 1 whilst all samples from Italy, Croatia and Serbia were in cluster 2. Of 70 samples from Scotland, 61 samples were in cluster 1, 6 in cluster 2 and only 2 in cluster 3; a similar distribution was seen for samples from France and Switzerland. Samples from England, Denmark and Wales were also predominantly found in cluster 1.

The admixture patterns were also examined in pooled individuals representing an independent set of 47 colony samples from Switzerland and the UK (Supplementary Table S10). The average *Q*-values estimated for the Swiss samples of *A*. *m*. *mellifera* varied between 0.145 ± 0.074 (protected) and 0.118 ± 0.042 (unprotected), which were lower than those inferred from a single individual (Supplementary Table S9). However, these estimates are not directly comparable as the pooled- and single-individual samples were from different apiaries. More comparable results were obtained for four colonies of variable ancestry from the UK that were simultaneously represented by a single worker and a pool of 16 workers. The *Q*-values inferred for each colony from the single worker and the pools were similar but always lower for the latter (Supplementary Table S10), a pattern that was also observed in the Swiss samples. This is an interesting finding that deserves to be fully investigated in a larger sample size.

## Discussion

The success of the numerous initiatives that are developing across Europe to protect and bring back the endangered dark honeybee rely on molecular tools capable of accurately detecting varying levels of C-derived introgression in a time- and cost-effective manner. In many conservation programs, the breeding stock has been routinely identified through wing morphometry and, more recently, through microsatellites^24^. However, inferring from data on Africanized honeybees^25^, wing morphometry is likely unable to detect low levels of C-lineage introgression into *A*. *m*. *mellifera*, a limitation that is overcome by microsatellites^11,12^. While adoption of microsatellites represented a major step in conservation management of *A*. *m*. *mellifera*^12^, it has been shown that a reduced number of high-graded SNPs^20^, outperform the multiallelic marker in estimating introgression^19,26^.

Here, from the 144 top-ranked SNPs, selected by their power in discriminating C- from M-lineage honeybees^20^, we designed, tested and validated four assays for genotyping with the iPLEX MassARRAY system. We provide the genomic information along with the PCR and iPLEX primers for 117 high-quality SNPs multiplexed in the four assays for immediate application in genetic surveys and conservation management of *A*. *m*. *mellifera*. In addition, we provide the dataset with the genotypes for haploid and diploid individuals of *A*. *m*. *mellifera*, *A*. *m*. *carnica* and *A*. *m*. *ligustica*, which can be used by others in introgression analysis as baseline reference populations with no need for inter-laboratory calibration^18^. As opposed to microsatellites, merging of SNP databases is straightforward as there are only two alleles per locus and different platforms will provide the same allele calls. If needed, curation will only involve SNP conversion from different platforms to be on the same DNA strand, which is much simpler than trying to harmonize different microsatellite allele sizes genotyped in different laboratories.

We show that C-lineage introgression can be accurately estimated from haploid, diploid, and combined haploid and diploid datasets (see Supplementary Information for details). These findings indicate that honeybee conservation managers can choose the software of their preference and, more importantly, can simultaneously analyse workers and drones without biasing estimates of C-lineage introgression in *A*. *m*. *mellifera* colonies.

The Assay Design software was able to combine only 127 of the 144 high-graded SNPs^20^ into four multiplexes. While the iPLEX protocol allows multiplexing up to 40 SNPs, only assay M1 (38 SNPs) approached the maximum plexing capacity. This is in part due to the relatively small size of the baseline SNP set from which the Assay Design had to work. However, the plex level of each assay can be expanded any time. By using the *Replex* option of the software, additional high-graded nuclear SNPs or even mitochondrial SNPs can be added to the customized four assays for detecting C-derived genes at both genetic compartments.

The iPLEX MassARRAY system revealed highly accurate and delivered high-quality calls for 117 of the 127 SNPs. Quality assessment was greatly facilitated by the honeybee haplodiploid system. Using the SNP calls of the drone subset, problematic SNPs were easily detected by locating genotypes erroneously typed heterozygous. Three such SNPs were consistently identified in numerous drones. While the mechanism responsible for the false allele is unclear, it is possible that gene homology is the source of miscalling at least in locus 1379-est5929. Using the 120-bp flanking region of this SNP locus, a NCBI query found a second hit with 98% similarity in the honeybee genome. The 117 SNPs were successfully genotyped in over 96% of the samples, indicating that the customized four assays and the iPLEX MassARRAY system work well in DNAs obtained from a variety of tissues with the virtually full spectrum of extraction methods routinely employed in honeybee research^27^.

The four combined SNP assays were able to estimate introgression with a high degree of accuracy. However, performance decreased to some extent when SNP assays were used singly and the 23-plex M4 showed the worst behaviour for most statistics. This finding is consistent with studies on other organisms which have also detected drops in accuracy when the number of SNPs is <25^28,29^. Further assessment of the four assays (used singly or combined) at the individual level indicates that there is a greater chance of misclassifying purebred individuals as admixed than the reverse, *viz*. misclassifying admixed individuals as purebred. This result has practical implications in conservation management suggesting that it is more likely that *A*. *m*. *mellifera* genetic diversity is erroneously discarded from the breeding population than C-derived genes are maintained. At this point, simulation and empirical studies are needed to determine the best threshold criterion to separate purebreds from admixed individuals^30^. While the stringent *Q*-value threshold of <0.05 arbitrarily established here for defining purebred *A*. *m*. *mellifera* may assure a more efficient purging of C-derived alleles, it may also lead to erosion of *A*. *m*. *mellifera* diversity and loss of unique gene complexes. The problem is that low diversity is particularly detrimental for honeybees because it may decrease colony resistance to brood diseases^31^ and increase genetic load at the sex locus^32^. Therefore, managers of *A*. *m*. *mellifera* conservatories need to make a trade-off between purging foreign alleles from the breeding population while minimizing the effects of reduced diversity.

Validation of the four SNP assays in an independent set of individuals, including F1 hybrids (obtained from controlled crosses purposely established for this study, as opposed to the simulated hybrids more commonly found in the literature), further confirms the resolution power of our customized SNP assays. Interestingly, the *Q*-values obtained for the F1 hybrids were in close proximity to the expected 0.50, although there was a bias towards C-derived genes as most *Q*-values were >0.50. When used singly, the SNP assays failed to correctly identify all purebred individuals and the *Q*-values were more dispersed around 0.50. However, when the assays were combined, the performance increased with all purebred individuals correctly classified and the *Q*-values showing a lower dispersion around 0.50. Interestingly, despite the lower number of SNPs contained in M1 + M3 (62 *vs* 117), this assay combination shows an overall performance similar to that of M1 + M2 + M3 + M4.

Sustainable conservation management requires tools capable of reliably identifying breeding colonies in a time- and cost-efficient manner. The SNP assays tested herein have a high resolution power for accurately estimating introgression, and the iPLEX MassARRAY system offers an interesting option for rapid and cost-effective genotyping. This system is very flexible and scalable allowing a variety of options for sample and assay throughput at a variable cost, depending on the chip format (24, 96, or 384) chosen. The 384 format, for example, allows genotyping 384 samples with a single assay at an approximate outsourced cost of 4.5€ per sample. Alternatively, this format could be used to genotype 192, 128, or 96 samples with two, three, or four assays, respectively. This option would incur in an increment of 4.5€ for any additional assay. Based on overall results, the best compromise between genotyping costs and assay accuracy is achieved when using M1 + M3.

Genotyping a single microsatellite multiplex in a 96-plate format costs approximately 2.5€ per sample. Introgression proportions using microsatellites has typically been estimated from over 11 loci, which requires genotyping a minimum of two multiplexes^11,24,26,33^ thereby doubling the per-sample cost. However, this charge does not include PCR and microsatellite fragment analysis. Contrary to microsatellites, outsourced SNP genotyping with the iPLEX MassArray system only requires DNA (instead of PCR products) to generate a table of genotypes ready to analyse, avoiding the hurdle of fragment analysis.

Honeybee queens mate in flight with up to 20 drones^34^. This means that in areas where *A*. *m*. *mellifera* and commercial colonies are sympatric, matings may occur with drones of C-lineage ancestry originating colonies made up of subfamilies with diverse genetic backgrounds. Although population-level studies typically require genotyping a single worker per colony^16^, colony-level introgression estimates may require genotyping several individuals to more effectively capture the colony structure. The problem is that genotyping several workers per colony is time consuming and costly. An economical way to circumvent this issue is to genotype pools instead of individuals^35^, provided that the genotyping system of choice is sensitive enough to detect low-frequency alleles.

Here, we assessed whether our customized SNPs assays and the iPLEX MassARRAY system offer a reliable alternative for pool genotyping. Both DNA and tissue pooling experiments show that the genotyping system is very sensitive as it was able to detect low frequency alleles. Despite the small number of SNPs showing consistent amplification across experiments, introgression analysis indicates that as few as 62 SNPs (M1 + M3) were able to detect highly diluted C-derived alleles. These results suggest that this system has the potential to detect C-lineage introgression in colonies with hybrid sub-families at low frequency, a scenario that might occur if drones of commercial colonies are able to accidentally enter congregation areas of conservatories.

Analysis of pool genotypes showed that miscalling was mainly due to the unequal contribution of each individual (different concentrations) and to the unbiased representation of allelic products that are present in a DNA pool, both common problems reported for DNA pools^35,36^. While pools constructed from equi-molar DNA concentrations would be the most correct approach to genotype a colony, pooling tissues is often the only option in conservation programs requiring screening of numerous colonies with a limited budget. Pooling tissue instead of DNA requires less time, effort and money during preparation in the laboratory and still enables detection of C-derived alleles even when most of the individuals in the pool are *A*. *m*. *mellifera*.

The introgression analysis on the samples collected throughout Europe and genotyped using the four SNP assays and the iPLEX MassARRAY system provides a rough picture of the genetic integrity of *A*. *m*. *mellifera*. This SNP survey adds to Pinto, *et al*.^13^ by expanding the sampling in France, Switzerland, UK and by including *de novo* Wales and Ireland. Concordant with earlier microsatellite^11,12^ and SNP^13,21^ surveys, C-lineage introgression in *A*. *m*. *mellifera* is heterogeneous across Europe. Samples originating from conservatories were generally less introgressed than those from unprotected areas. Our previous and this SNP survey revealed that Scotland, Norway, Netherlands and now Ireland possess important pockets of pure *A*. *m*. *mellifera*. Ireland represents a particularly interesting case of *A*. *m*. *mellifera* diversity because, contrary to the other countries, the survey was performed in unprotected populations from a wide geographical area.

As this and previous studies^11–13,21^ represent only partial, and in some cases biased, assessments on the status of the genetic integrity of *A*. *m*. *mellifera* across its distributional range, this novel tool now makes it possible to perform a comprehensive genetic survey in a time- and cost-efficient manner. We suggest that if the efficacy of this SNP tool is generally agreed among stakeholders the next step is for them to seek input from government agencies and/or research facilities and begin to describe the purity of their honeybee populations on as wide a geographic area as possible in order that conservation efforts correctly and efficiently target regions of greatest concern and greatest possible reward.

## Methods

### Assay design

Muñoz, *et al*.^20^ identified 144 highly informative SNPs for estimating C-lineage introgression in *A*. *m*. *mellifera*. The flanking regions (60 bp of either side) of these SNPs were used to design multiplexed assays with the software Assay Design 4.0 (Agena BioScience^TM^) for genotyping using the Agena BioScience iPLEX chemistry and the MassARRAY® MALDI-TOF platform (hereafter abbreviated to iPLEX MassARRAY). The software searched for optimal areas within the 120-bp flanking regions to design forward and reverse PCR primers while constructing the different multiplexes. The maximum multiplexing capacity (40 SNPs) allowed by the iPLEX chemistry was attempted whilst preventing hairpin and dimer formation. In addition to the PCR primers, the software designed the iPLEX extension primer placed immediately adjacent to each SNP. Of the 144 SNPs, the Assay Design was able to combine 127 SNPs distributed along four multiplexed assays (see Supplementary Table S1 for sequences of the flanking regions, sequences of PCR and iPLEX reaction primers, and composition of the four multiplexes). The putative functional role of the genes marked by each SNP was identified using SNPeff 4.3 tool build^37^ and the NCBI *Apis mellifera* annotation genome version 102^38^.

A wide array of analyses were carried out to validate the SNP assays and to assess (i) their accuracy when genotyped with the MassARRAY system, (ii) their performance when employed individually or combined, (iii) and their sensitivity when employed in pools of DNA and tissue. To that end, different combinations of samples, representing single and pooled, haploid and diploid individuals were used, as depicted in Fig. 2 and detailed in each section below.

### Samples and DNA extraction

A total of 464 colonies (represented by a single haploid drone, a single diploid worker, multiple workers, or pools of drones or workers; Supplementary Table S2) were sampled across Europe (Fig. 1). The samples originated from colonies in the (i) *A*. *m*. *mellifera* (N = 462) native range in Western and Northern Europe (protected and unprotected areas), (ii) *A*. *m*. *ligustica* (N = 10), and *A*. *m*. *carnica* native ranges (N = 10) in South-eastern Europe, (iii) introduced range of *A*. *m*. *carnica* in Switzerland (N = 8), Germany (Kirchhain; N = 16) and Scotland (N = 3), (iv) commercial strain Buckfast from Switzerland, Scotland, and Denmark (N = 11), and (v) F1 hybrid crosses performed in isolated mating stations in Denmark (N = 19). Nine samples of *A*. *m*. *carnica* and seven *A*. *m*. *ligustica*, previously genotyped using the GoldenGate® Assay in the BeadArray platform of Illumina^13^, were added to the dataset to have a better representation of C-lineage.

Genomic DNA was extracted from the head, antennae, thorax (entire or ~half), legs, or abdomen of adults or immatures (larvae or pupae) of a single individual, multiple individuals (extracted, then pooled), or a pool of individuals (mixed tissue, then extracted) per colony in 561 samples (Supplementary Table S2). The extraction methods included phenol-chloroform, CTAB, commercial kits (Qiagen EZ1 DNA tissue kit, Omega bio-tek EZNA kit), and magnetic beads using the KingFisher™ Flex Purification System. These represent the wide array of tissues and extraction methods commonly used in honeybee research^27^. The DNA samples were set at a concentration of 10–15 ng/µl and sent to *Instituto Gulbenkian de Ciência* (Portugal) for SNP genotyping.

### SNP genotyping and quality control

A total of 573 samples (561 plus 12 DNA pools, Supplementary Table S2) were genotyped for the 127 SNP loci multiplexed in the four assays using the iPLEX chemistry and the MassARRAY® MALDI-TOF genotyping platform^39^. The genotypes generated for the 573 samples (Supplementary Table S11) were subjected to quality control filters to discard SNP loci and samples with poor or inconsistent amplification. SNPs and samples with missing data >20% (Supplementary Table S1) and >30% (Supplementary Table S2), respectively, were excluded from the dataset (Supplementary Table S3).

### Assessing genotyping accuracy

The genotyping accuracy was assessed on the subset of single haploid drones of *A*. *m*. *mellifera* (N = 103), *A*. *m*. *ligustica* (N = 10) and *A*. *m*. *carnica* (N = 15), by (i) identifying the heterozygous SNP loci (N = 128; Fig. 2 and Supplementary Table S2) and (ii) comparing the SNP calls generated for a variable number of individuals by the iPLEX MassARRAY system with those obtained with the GoldenGate® Assay genotyped in the BeadArray platform of Illumina (N = 96 individuals^13^) and with the HiSeq. 2500 platform of Illumina (N = 32 individuals; see whole-genome sequencing details in Parejo, *et al*.^21^ and Henriques, *et al*.^40^). The SNP loci that were called heterozygous by the MassARRAY system in >10% of the drones and showed inconsistent genotypes between at least two genotyping technologies in >5% of the drones were excluded from further analysis (Supplementary Tables S1 and S3).

### Introgression estimation

Introgression proportions (*Q*-values) were estimated by ADMIXTURE^41^ using datasets of varying ploidies (haploids, diploid, and their combination), which produced similar *Q*-values (see Supplementary Information for details). *Q*-values were estimated for K = 2 using 10,000 iterations in 20 independent runs. The convergence between iterations was monitored by comparing log-likelihood scores (LLS) using the default termination criterion set to stop when LLS increases by <0.0001 between iterations. CLUMPAK^42^ was used to summarize and visualize the *Q*-plots.

### Assessing performance of the SNP assays

The performance of the SNP assays in estimating C-lineage introgression in *A*. *m*. *mellifera* was assessed by comparing the *Q*-values inferred by them with those inferred from 2.399 million SNPs identified in WGs (see Parejo, *et al*.^21^ and Henriques, *et al*.^40^ for further details). A total of 38 drones (4 *A*. *m*. *ligustica*, 7 *A*. *m*. *carnica*, 11 purebred *A*. *m*. *mellifera*, and 16 admixed *A*. *m*. *mellifera*), for which there were WG sequence data available, was used in this comparison (Fig. 2). The 4 *A*. *m*. *ligustica* and 2 of the 7 *A*. *m*. *carnica* previously genotyped using the GoldenGate® Assay^13^ were added to this step for a better representation of lineage C. The performance of the four assays (individually or combined) was assessed by (i) Pearson’s correlation coefficient (*r*), (ii) similarity score obtained by CLUMPAK, (iii) absolute accuracy error calculated as the absolute difference between *Q*-values inferred from the SNP assays and the 2.399 million SNPs, (iv) mean accuracy calculated via percentage of absolute error, (v) absolute precision error calculated via standard deviation of the absolute differences, (vi) number of purebred individuals classified as admixed, and (vii) number of admixed individuals classified as purebred. Admixed individuals were defined by a threshold *Q*-value > 0.05. Any individual with *Q*-value between 0 and <0.05 or >0.95 and 1 was classified as purebred *A*. *m*. *mellifera* and C-lineage (*A*. *m*.*carnica* or *A*. *m*. *ligustica*), respectively.

### Validating the SNP assays

The four assays were validated and tested using an independent subset of 62 workers, including 30 *A*. *m*. *mellifera* (Endelave, Denmark), 16 *A*. *m*. *carnica* (Kirchhain, Germany), and 16 F1 hybrids obtained from crosses between *A*. *m*. *mellifera* queens, from the conservatory in Læsø, and *A*. *m*. *carnica* drones from Mandø, Denmark (Fig. 2 and Supplementary Table S12). The crosses were performed in the isolated mating station of Mandø in 2016. *Q*-values were inferred from the four assays (individually or combined) by ADMIXTURE and then compared with the defined thresholds of >0.95 for *A*. *m*. *carnica*, <0.05 for *A*. *m*. *mellifera*, and ~0.5 for the F1 hybrids.

### Assessing sensitivity of the MassARRAY system in pooled DNA

Pools of tissue or DNA are a cost-efficient option for estimating introgression in organisms with a polyandrous mating system like the honeybee. However, pooling can only be adopted if the genotyping system is able to consistently detect low-frequency alleles. The sensitivity of the MassARRAY system was assessed in a dilution experiment of varying ratios of DNAs of two haploid drones: one *A*. *m*. *ligustica* and one *A*. *m*. *mellifera* (Fig. 2). The two drones displayed the highest number of alternate alleles for the 127 highly-informative SNPs identified in a large dataset previously genotyped with the GoldenGate® Assay^13^.

The experiment was performed by pooling the DNA of the two drones using volume ratios of 10:20, 5:20, 2:20, 1:20, and 0.5:20 *A*. *m*. *ligustica* to *A*. *m*. *mellifera* (Fig. 2). The number of replicates was three for 1:20 and 0.5:20 and two for the remaining ratios, as they were nested in the higher dilution factors. The pools were genotyped for the four assays using the iPLEX MassARRAY. The genotypes generated from the pooled DNAs were compared with those expected and the number of mismatches was recorded. The expected genotypes of the pools were inferred from the SNP calls for the single drones.

The sensitivity of the genotyping system in detecting C-lineage ancestry in the pooled samples was also assessed via introgression analysis. The *Q*-values were estimated by ADMIXTURE for each DNA pool using the expected and called genotypes for a variable number of SNPs (four assays and best assay combination, as defined by *r*).

### Assessing sensitivity of the MassARRAY system in pooled tissue

The sensitivity of the MassARRAY system was further assessed in tissue pools (Supplementary Table S12). A total of 22 pools were constructed using varying ratios of workers (1:1, 1:2, 1:3, 1:7) of two different ancestries chosen among *A*. *m*. *mellifera* (N = 30), A. *m*. *carnica* (N = 16), Buckfast (N = 3), and F1 hybrids (*A*. *m*. *mellifera* queens x *A*. *m*. *carnica* drones; N = 19), as detailed in Fig. 2 and Supplementary Table S13. The DNA was extracted twice (individually and pooled) from the thorax, which had been cut in two identical portions. The DNA concentrations of individual and pooled extractions were measured using NanoDrop^TM^ (Supplementary Table S12).

The sensitivity of the genotyping system was first assessed by comparing the SNP calls obtained for the single workers with those obtained for the pools of workers. Mismatches were counted and the error identified among the following sources: (i) pools displayed alleles uncalled in single workers and *vice versa*, (ii) SNP calls of the pools matched those of the worker with higher DNA concentration, (iii) SNP calls of the pools matched the most frequent allele, and (iv) the least frequent allele. The sensitivity of the genotyping system in detecting C-lineage ancestry in the different pools was also assessed via introgression analysis. The *Q*-values were estimated for the 22 pools from the expected and called genotypes, for a variable number of SNPs (four assays and best assay combination), using ADMIXTURE. The expected genotypes were inferred from the calls obtained for the single workers.

### Applying the SNP assays

The four assays were used to genotype in the MassARRAY platform 462 samples representing *A*. *m*. *mellifera* (N = 425), *A*. *m*. *ligustica* (N = 10), *A*. *m*. *carnica* (N = 21), and Buckfast (N = 6) from 8 13 European countries (Figs 1 and 2). Samples of *A*. *m*. *mellifera* originated from protected (N = 125) and unprotected (N = 300) areas. Of the 462 samples, 415 were represented by a single individual and 47 by pooled individuals (16 pooled workers from colonies of *A*. *m*. *mellifera*, *A*. *m*. *carnica* and Buckfast; 30 pooled drones from colonies of *A*. *m*. *mellifera*; Supplementary Table S2). Additionally, a subset of four colonies (two *A*. *m*. *mellifera*, one *A*. *m*. *carnica*, and one Buckfast) from Scotland and England was represented by both a pool of 16 workers and one individual worker. For a better C-lineage representation, nine samples of *A*. *m*. *carnica* and 7 of *A*. *m*. *ligustica* (each representing a single individual and colony), previously genotyped using the GoldenGate® Assay^13^, were added to the dataset. *Q*-values were inferred from the genotypes of single and pooled samples using ADMIXTURE.

The genotype data were further examined by network analysis using the software Graphia Professional (Kajeka Ltd, Edinburgh, UK). For each sample, SNPs were scored 0 when same as reference (*A*. *m*. *carnica*), 1 for heterozygous and 2 for homozygous different to reference, i.e. representing the *A*. *m*. *mellifera* allele. Where data was missing, the SNP was scored 1.01. For ease of interpretation, the total combined score for each SNP in each sample was calculated and the SNPs reordered from the smallest score to the largest. The SNP data and associated sample metadata was loaded into Graphia and a Pearson correlation matrix was calculated comparing the profile of SNP scores for each sample. A network graph was then constructed by connecting the nodes (samples) with edges (where the correlation exceeded the threshold value *r* > 0.27). Utilising the overlay of metadata the graph was then explored and clustered using the Markov Cluster (MCL) algorithm^43^ at an inflation value (which determines cluster granularity) of 1.2.

### Data availability

*A*. *m*. *carnica* and *A*. *m*. *mellifera* whole-genome sequence data is deposited at the ENA (www.ebi.ac.uk/ena) under study accession number PRJEB16533.

## Electronic supplementary material

Supplementary Tables

Supplementary Information
