# Supplementary material for: High sample throughput genotyping for estimating C-lineage introgression in the dark honeybee: an accurate and cost-effective SNP-based tool
Source: Sci Rep. 2018 Jun 4;8:8552. doi: 10.1038/s41598-018-26932-1 (PMC5986779; doi:10.1038/s41598-018-26932-1)
Supplement: Supplementary file 2 — Supplementary Information [file 41598_2018_26932_MOESM2_ESM.pdf]

Supplementary Information

High sample throughput genotyping for estimating C-lineage introgression in the dark honeybee: an accurate and cost-effective SNP-based tool

Dora Henriques[1,2], Keith A. Browne[3], Mark W. Barnett[4], Melanie Parejo[5], Per Kryger[6], Tom C. Freeman[4], Irene Muñoz[7], Lionel Garnery[8,9], Fiona Highet[10], J. Spencer Jonhston[11], Grace P. McCormack[3], M. Alice Pinto[1*]

[1]Mountain Research Centre (CIMO), Polytechnic Institute of Bragança, 5300-253 Bragança, Portugal

[2]Centre of Molecular and Environmental Biology (CBMA), University of Minho, Campus de Gualtar, 4710-057 Braga, Portugal

[3] Department of Zoology, Ryan Institute, School of Natural Sciences, National University of Ireland Galway, Galway, Ireland

[4]The Roslin Institute and Royal (Dick) School of Veterinary Studies, University of Edinburgh, Easter Bush, Edinburgh, Midlothian, EH25 9RG, Scotland, UK

[5]Agroscope, Swiss Bee Research Centre, 3003 Bern, Switzerland

[6]Aarhus University, Department of Agroecology, Slagelse, 4200, Denmark

[7]Área de Biología Animal, Dpto. de Zoología y Antropología Física, Universidad de Murcia, Campus de Espinardo, 30100 Murcia, Spain

[8]Laboratoire Evolution, Génomes et Spéciation, CNRS, Gif-sur-Yvette, France

[9]Saint Quentin en Yvelines, Université de Versailles, Versailles, France

[10]Science and Advice for Scottish Agriculture (SASA), Roddinglaw Road, Edinburgh, EH12 9FJ, Scotland, UK

[11]Department of Entomology, Texas A&M University, College Station, USA

Supplementary Figure S1

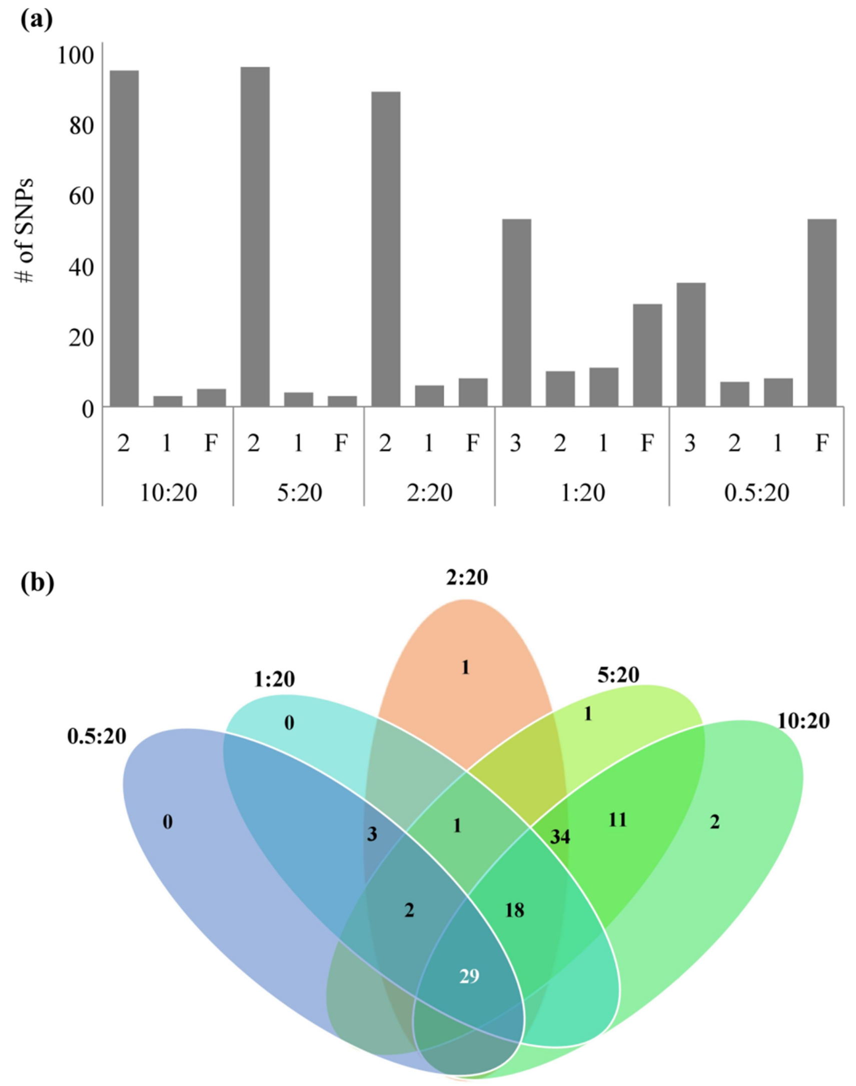

Supplementary Figure S1. Sensitivity of the MassARRAY genotyping system in detecting *A. m. ligustica* alleles in DNA pools of *A. m. ligustica* and *A. m. mellifera*. **(a)** The sensitivity was assessed by counting the number of SNPs that were correctly called in either 1, 2, or 3 replicates or that failed (F) in all replicates for that pool (dilution ratios of 10:20, 5:20, 2:20, 1:20, 0.5:20). While only 7 SNPs failed detecting the *A. m. ligustica* allele in the 10:20 pool, that number increased with dilution of the *A. m. ligustica* DNA, being 56 for the 0.5:20 pool. **(b)** Venn diagram of the number of SNP loci with 100% successful SNP calls at each dilution ratio. The central overlap shows the 29 SNPs that resulted in 100% success for all dilution ratios.

Comparing approaches of introgression estimation

The clustering approach implemented by the software packages STRUCTURE[1] and ADMIXTURE[2] has been preferred for estimating introgression (*Q*-values) in honeybees[3], especially of C-lineage in *A. m. mellifera*[4-7]. While both packages handle haploid and diploid data, it is unclear whether *Q*-values are accurate for datasets combining different ploidies, a circumstance that might occur if drones and workers are required to be genotyped. To support the decision making of honeybee managers, the consistency of the *Q*-values was assessed by comparing the outputs of ADMIXTURE with those of STRUCTURE.

Methods

The *Q*-values were assessed by comparing (i) the output of ADMIXTURE and STRUCTURE generated from a subset of haploid drones (N=112), and (ii) the output of ADMIXTURE generated from diploid workers (N=112) and combined drones and workers (N=224). Differences in *Q*-values were assessed by Mann-Whitney test.

In STRUCTURE, *Q*-values were estimated for two ancestral clusters (K=2) using the admixture ancestry and correlated allele frequency models with the unsupervised option. The initial burn-in was set to 250,000 followed by 750,000 Markov chain Monte Carlo iterations. Over 20 independent runs were performed to confirm consistency across runs. In ADMIXTURE, *Q*-values were also estimated for K=2 using 10,000 iterations in 20 independent runs. The convergence between iterations was monitored by comparing log-likelihood scores (LLS) using the default termination criterion set to stop when LLS increases by <0.0001 between iterations. CLUMPAK [43] was used to summarize and visualize the STRUCTURE and ADMIXTURE *Q*-plots.

Results and discussion

As expected, the two software packages estimated virtually the same *Q*-values (*P*-value=0.89, Mann-Whitney test; Table 1). Similar *Q*-values were also inferred from haploid, diploid, and combined haplodiploid datasets (*P*-value>0.87, Mann-Whitney test; Table 2).

We show that C-lineage introgression can be accurately estimated from haploid, diploid, and combined haploid and diploid datasets using either STRUCTURE or ADMIXTURE. These findings indicate that honeybee conservation managers can choose the software of their preference and, more importantly, can simultaneously analyse workers and drones without biasing estimates of C-lineage introgression in *A. m. mellifera* colonies.

Table 1. *Q*-values inferred from the four assays (117 SNPs) by STRUCTURE and ADMIXTURE for 112 drones, of which 96 were genotyped in the MassARRAY® MALDI-TOF platform and 16 (9 *A. m. carnica* and 7 *A. m. ligustica*) were previously genotyped using the GoldenGate® Assay in the Illumina's BeadArray platform (see Pinto, et al. [6]) and added to the dataset for a better representation of lineage C.

Sample ID	STRUCTURE	ADMIXTURE
2680	0.987	1.000
2681	0.984	1.000
2682	0.986	1.000
2683	0.992	1.000
2684	0.968	1.000
2685	0.963	1.000
2686	0.973	1.000
2687	0.965	0.996
2688	0.941	0.958
2689	0.989	1.000

2690	0.998	1.000
2691	0.991	1.000
2692	0.997	1.000
2693	0.975	1.000
2694	0.994	1.000
2695	0.998	1.000
2696	0.977	1.000
2698	0.634	0.641
2701	0.592	0.599
2702	0.491	0.495
2703	0.243	0.239
2704	0.009	0.000
2721	0.993	1.000
2722	0.996	1.000
2723	0.965	1.000
2724	0.972	1.000
2725	0.976	1.000
2726	0.987	1.000
2730	0.977	1.000
2731	0.951	0.979
2732	0.969	1.000
2733	0.996	1.000
2734	0.988	1.000
2735	0.954	0.984
2736	0.943	0.962
2738	0.970	1.000
2739	0.991	1.000
2740	0.998	1.000
2741	0.978	1.000
2742	0.990	1.000
2743	0.996	1.000
2744	0.205	0.200
2745	0.341	0.341
2746	0.169	0.165
2747	0.119	0.105
2748	0.081	0.073
2756	0.024	0.013
2757	0.112	0.108
2758	0.142	0.134
2760	0.187	0.180
2768	0.002	0.000
2769	0.003	0.000
2770	0.010	0.000
2771	0.008	0.000

2772	0.015	0.000
2773	0.076	0.061
2774	0.154	0.144
2775	0.132	0.126
2776	0.088	0.068
2777	0.003	0.000
2778	0.049	0.041
2779	0.002	0.000
2780	0.016	0.000
2781	0.002	0.000
2782	0.008	0.000
2783	0.016	0.000
2784	0.008	0.000
2785	0.003	0.000
2794	0.004	0.000
2795	0.078	0.070
2796	0.003	0.000
2797	0.193	0.185
2798	0.082	0.076
2799	0.002	0.000
2800	0.009	0.000
2801	0.002	0.000
2802	0.002	0.000
2803	0.009	0.000
2811	0.014	0.000
2812	0.009	0.009
2813	0.017	0.018
2814	0.052	0.044
2815	0.008	0.000
2816	0.536	0.540
2817	0.002	0.000
2820	0.018	0.003
2821	0.006	0.000
2822	0.002	0.000
2823	0.008	0.000
2824	0.006	0.000
2825	0.009	0.000
2826	0.090	0.083
2827	0.018	0.017
2828	0.340	0.341
2829	0.144	0.142
2830	0.039	0.025
2831	0.299	0.299
2832	0.055	0.039

2833	0.121	0.114
2834	0.010	0.000
2835	0.083	0.074
2836	0.068	0.055
2837	0.044	0.029
2838	0.030	0.019
2895	0.125	0.121
2896	0.237	0.236
2897	0.219	0.220
2898	0.130	0.119
2899	0.128	0.118
2900	0.357	0.358
2901	0.192	0.191
2902	0.192	0.186

Table 2. *Q*-values inferred from the four assays (117 SNPs) by ADMIXTURE for haploid drones (N=112), diploid workers (N=112), and their combination (N=224). Of the 112 drones, 96 were genotyped in the MassARRAY® MALDI-TOF platform and 16 (9 *A. m. carnica* and 7 *A. m. ligustica*) were previously genotyped using the GoldenGate® Assay in the Illumina's BeadArray platform (see Pinto, et al.[6]) and added to the dataset for a better representation of lineage C.

Sample ID	Ploidy	*Q*-values	
		Individual datasets	Combined datasets
2680	Haploid	1.000	1.000
2681	Haploid	1.000	1.000
2682	Haploid	1.000	1.000
2683	Haploid	1.000	1.000
2684	Haploid	1.000	1.000
2685	Haploid	1.000	0.999
2686	Haploid	1.000	1.000
2687	Haploid	0.996	1.000
2688	Haploid	0.958	0.969
2689	Haploid	1.000	1.000
2690	Haploid	1.000	1.000

2691	Haploid	1.000	1.000
2692	Haploid	1.000	1.000
2693	Haploid	1.000	1.000
2694	Haploid	1.000	1.000
2695	Haploid	1.000	1.000
2696	Haploid	1.000	1.000
2698	Haploid	0.641	0.647
2701	Haploid	0.599	0.603
2702	Haploid	0.495	0.499
2703	Haploid	0.239	0.236
2704	Haploid	0.000	0.000
2721	Haploid	1.000	1.000
2722	Haploid	1.000	1.000
2723	Haploid	1.000	1.000
2724	Haploid	1.000	1.000
2725	Haploid	1.000	1.000
2726	Haploid	1.000	1.000
2730	Haploid	1.000	0.983
2731	Haploid	0.979	1.000
2732	Haploid	1.000	0.979
2733	Haploid	1.000	1.000
2734	Haploid	1.000	1.000
2735	Haploid	0.984	0.986
2736	Haploid	0.962	0.991
2738	Haploid	1.000	1.000
2739	Haploid	1.000	1.000
2740	Haploid	1.000	1.000
2741	Haploid	1.000	1.000
2742	Haploid	1.000	1.000
2743	Haploid	1.000	1.000
2744	Haploid	0.200	0.200
2745	Haploid	0.341	0.345
2746	Haploid	0.165	0.166
2747	Haploid	0.105	0.114
2748	Haploid	0.073	0.074
2756	Haploid	0.013	0.017
2757	Haploid	0.108	0.110
2758	Haploid	0.134	0.139
2760	Haploid	0.180	0.182
2768	Haploid	0.000	0.000
2769	Haploid	0.000	0.000
2770	Haploid	0.000	0.000
2771	Haploid	0.000	0.000
2772	Haploid	0.000	0.000

2773	Haploid	0.061	0.066
2774	Haploid	0.144	0.139
2775	Haploid	0.126	0.127
2776	Haploid	0.068	0.073
2777	Haploid	0.000	0.000
2778	Haploid	0.041	0.045
2779	Haploid	0.000	0.000
2780	Haploid	0.000	0.000
2781	Haploid	0.000	0.000
2782	Haploid	0.000	0.000
2783	Haploid	0.000	0.000
2784	Haploid	0.000	0.000
2785	Haploid	0.000	0.000
2794	Haploid	0.000	0.000
2795	Haploid	0.070	0.066
2796	Haploid	0.000	0.000
2797	Haploid	0.185	0.187
2798	Haploid	0.076	0.074
2799	Haploid	0.000	0.000
2800	Haploid	0.000	0.000
2801	Haploid	0.000	0.000
2802	Haploid	0.000	0.000
2803	Haploid	0.000	0.000
2811	Haploid	0.000	0.000
2812	Haploid	0.009	0.000
2813	Haploid	0.018	0.000
2814	Haploid	0.044	0.046
2815	Haploid	0.000	0.000
2816	Haploid	0.540	0.548
2817	Haploid	0.000	0.000
2820	Haploid	0.003	0.006
2821	Haploid	0.000	0.000
2822	Haploid	0.000	0.000
2823	Haploid	0.000	0.000
2824	Haploid	0.000	0.000
2825	Haploid	0.000	0.000
2826	Haploid	0.083	0.085
2827	Haploid	0.017	0.000
2828	Haploid	0.341	0.343
2829	Haploid	0.142	0.141
2830	Haploid	0.025	0.033
2831	Haploid	0.299	0.302
2832	Haploid	0.039	0.046
2833	Haploid	0.114	0.116

2834	Haploid	0.000	0.000
2835	Haploid	0.074	0.072
2836	Haploid	0.055	0.057
2837	Haploid	0.029	0.014
2838	Haploid	0.019	0.029
2895	Haploid	0.121	0.121
2896	Haploid	0.236	0.238
2897	Haploid	0.220	0.222
2898	Haploid	0.119	0.118
2899	Haploid	0.118	0.117
2900	Haploid	0.358	0.362
2901	Haploid	0.191	0.188
2902	Haploid	0.186	0.184
17317	Diploid	1.000	0.909
17316	Diploid	1.000	0.909
17303	Diploid	0.977	0.844
17300	Diploid	0.975	0.879
17306	Diploid	1.000	0.896
17339	Diploid	0.994	0.884
17336	Diploid	1.000	0.925
17335	Diploid	1.000	0.878
17334	Diploid	1.000	0.946
17296	Diploid	1.000	0.902
17290	Diploid	1.000	0.928
17293	Diploid	1.000	0.910
17320	Diploid	1.000	0.899
17321	Diploid	1.000	0.914
17340	Diploid	0.956	0.876
17341	Diploid	0.943	0.867
20_1	Diploid	0.000	0.012
20_10	Diploid	0.000	0.015
20_11	Diploid	0.022	0.020
20_12	Diploid	0.000	0.000
20_14	Diploid	0.000	0.019
20_15	Diploid	0.000	0.000
20_17	Diploid	0.125	0.135
20_19	Diploid	0.475	0.442
20_2	Diploid	0.005	0.024
20_22	Diploid	0.000	0.000
20_23	Diploid	0.000	0.000
20_25	Diploid	0.000	0.000
20_27	Diploid	0.036	0.038
20_29	Diploid	0.000	0.002
20_3	Diploid	0.014	0.015

20_32	Diploid	0.000	0.000
20_33	Diploid	0.044	0.043
20_34	Diploid	0.000	0.000
20_36	Diploid	0.000	0.000
20_37	Diploid	0.055	0.062
20_5	Diploid	0.000	0.014
20_6	Diploid	0.068	0.071
20_8	Diploid	0.000	0.000
20_9	Diploid	0.102	0.113
45_11	Diploid	0.297	0.284
45_12	Diploid	0.304	0.287
45_13	Diploid	0.096	0.101
45_14	Diploid	0.041	0.068
45_15	Diploid	1.000	0.951
45_16	Diploid	0.948	0.895
45_17	Diploid	0.029	0.054
45_18	Diploid	0.912	0.855
45_19	Diploid	0.482	0.451
45_20	Diploid	0.953	0.889
45_21	Diploid	0.108	0.112
45_22	Diploid	0.000	0.000
45_23	Diploid	0.039	0.050
45_24	Diploid	0.350	0.326
45_25	Diploid	0.043	0.054
45_26	Diploid	0.104	0.108
45_27	Diploid	0.047	0.072
45_28	Diploid	0.025	0.044
45_39	Diploid	0.489	0.455
45_45	Diploid	0.938	0.868
45_48	Diploid	0.945	0.906
45_49	Diploid	0.925	0.889
45_55	Diploid	0.638	0.595
45_92	Diploid	0.649	0.604
78_169	Diploid	0.043	0.054
78_170	Diploid	0.063	0.080
78_171	Diploid	0.088	0.100
78_173	Diploid	0.090	0.103
78_174	Diploid	0.135	0.141
78_175	Diploid	0.052	0.071
78_177	Diploid	0.022	0.036
78_178	Diploid	0.189	0.197
78_180	Diploid	0.570	0.522
78_181	Diploid	0.019	0.044
78_184	Diploid	0.014	0.028

78_185	Diploid	0.052	0.076
78_186	Diploid	0.194	0.196
78_187	Diploid	0.032	0.061
78_189	Diploid	0.000	0.030
78_191	Diploid	0.000	0.022
78_192	Diploid	0.062	0.073
78_194	Diploid	0.490	0.465
78_195	Diploid	0.082	0.095
78_196	Diploid	0.162	0.159
78_197	Diploid	0.143	0.147
78_199	Diploid	0.081	0.092
78_201	Diploid	0.055	0.066
78_202	Diploid	0.138	0.152
61_421	Diploid	0.324	0.308
61_422	Diploid	0.387	0.363
61_423	Diploid	0.382	0.361
61_424	Diploid	0.322	0.306
61_425	Diploid	0.252	0.237
61_426	Diploid	0.167	0.168
61_427	Diploid	0.261	0.251
61_428	Diploid	0.246	0.235
61_429	Diploid	0.229	0.223
61_430	Diploid	0.250	0.244
61_431	Diploid	0.291	0.277
61_432	Diploid	0.278	0.277
61_433	Diploid	0.607	0.558
61_434	Diploid	0.565	0.523
61_435	Diploid	0.350	0.335
61_436	Diploid	0.491	0.464
61_437	Diploid	0.382	0.366
61_438	Diploid	0.219	0.208
61_439	Diploid	0.245	0.240
61_440	Diploid	0.087	0.091
61_441	Diploid	0.200	0.193
61_442	Diploid	0.240	0.237
61_443	Diploid	0.159	0.159
61_444	Diploid	0.160	0.153

References

1 Pritchard, J. K., Stephens, M. & Donnelly, P. Inference of Population Structure Using Multilocus Genotype Data. *Genetics* **155**, 945-959 (2000).

2 Alexander, D. H., Novembre, J. & Lange, K. Fast model-based estimation of ancestry in unrelated individuals. *Genome Research* **19**, 1655-1664 (2009).

3 Meixner, M. D. *et al.* Standard methods for characterising subspecies and ecotypes of *Apis mellifera*. *Journal of Apicultural Research* **52**, 1-28 (2013).

4 Jensen, A. B., Palmer, K. A., Boomsma, J. J. & Pedersen, B. V. Varying degrees of *Apis mellifera ligustica* introgression in protected populations of the black honeybee, *Apis mellifera mellifera*, in northwest Europe. *Molecular Ecology* **14**, 93-106 (2005).

5 Soland-Reckeweg, G., Heckel, G., Neumann, P., Fluri, P. & Excoffier, L. Gene flow in admixed populations and implications for the conservation of the Western honeybee, *Apis mellifera*. *Journal of Insect Conservation* **13**, 317, doi:10.1007/s10841-008-9175-0 (2008).

6 Pinto, M. A. *et al.* Genetic integrity of the Dark European honeybee (*Apis mellifera mellifera*) from protected populations: a genome-wide assessment using SNPs and mtDNA sequence data. *Journal of Apicultural Research* **53**, 269-278, doi:10.3896/ibra.1.53.2.08 (2014).

7 Parejo, M. *et al.* Using Whole-Genome Sequence information to foster conservation efforts for the european dark honeybee, *Apis mellifera mellifera*. *Frontiers in Ecology and Evolution* **4**, doi:10.3389/fevo.2016.00140 (2016).
